# Prescribing Practices, Polypharmacy, and Drug Interaction Risks in Anticoagulant Therapy: Insights from a Secondary Care Hospital

**DOI:** 10.3390/jcm15020800

**Published:** 2026-01-19

**Authors:** Javedh Shareef, Sathvik Belagodu Sridhar, Shadi Ahmed Hamouda, Ahsan Ali, Ajith Cherian Thomas

**Affiliations:** 1 Department of Clinical Pharmacy & Pharmacology, RAK College of Pharmacy, RAK Medical & Health Sciences University, Ras Al Khaimah 11172, United Arab Emirates; 2 Ibrahim Bin Hamad Obaidallah Hospital, Emirates Health Services, Ras Al Khaimah 11172, United Arab Emirates; shadi.ahmed@ehs.gov.ae; 3 Internal Medicine, Ibrahim Bin Hamad Obaidallah Hospital, Emirates Health Services, Ras Al Khaimah 11172, United Arab Emirates; ahsan.ali5@nhs.net (A.A.); drajitct@gmail.com (A.C.T.)

**Keywords:** anticoagulants, prescriptions, polypharmacy, drug interactions, comorbidity

## Abstract

**Background/Objectives**: Blood thinners (anticoagulants) remain the first line pharmacotherapy for the management of cardiovascular and thromboembolic disorders. The increased utilization of polypharmacy, likely driven by the greater burden of comorbidities, elevates the risk of potential drug–drug interactions (pDDIs) and creates a significant challenge in anticoagulant management. The aim of the study was to assess the prescribing trend and impact of polypharmacy and pDDIs in patients receiving anticoagulant drug therapy in a public hospital providing secondary care. **Methods**: A cross-sectional observational study was undertaken between January–June 2023. Data from electronic medical records of prescriptions for anticoagulants were collected, analyzed for prescribing patterns, and checked for pDDIs using Micromedex database 2.0^®^. Utilizing binary logistic regression, the relationship between polypharmacy and sociodemographic factors was assessed. Multivariate logistic regression analysis served to uncover determinants linked to pDDIs. **Results:** Of the total 130 patients, females were predominant (58.46%), with a higher prevalence among those aged 61–90 years. Atrial fibrillation emerged as the main clinical reason and apixaban (51.53%) ranked as the top prescribed anticoagulant in our cohort. Among the 766 pDDIs identified, the majority [401 (52.34%)] were categorized as moderate in severity. Polypharmacy was strongly linked to age (*p* = 0.001), the Charlson comorbidity index (CCI) (*p* = 0.040), and comorbidities (*p* = 0.005) in the binary logistic regression analysis. In the multivariable analysis, the number of medications remain a strong predictor of pDDIs (adjusted OR: 30.514, *p* = 0.001). **Conclusions**: Polypharmacy and pDDIs were exhibited in a significant segment of cohort receiving anticoagulant therapy, with strong correlations to age, CCI, comorbidities, and the number of medications. A multidimensional approach involving collaboration among healthcare providers assisted by clinical decision support systems can help optimize the management of polypharmacy, minimize the risks of pDDIs, and ultimately enhance health outcomes.

## 1. Introduction

Anticoagulants contribute substantially in clinical settings, markedly in hospitals, in the management of cardiovascular and thromboembolic disorders [1,2]. The common agents include warfarin, direct oral anticoagulants (DOACs) such as rivaroxaban, dabigatran, and apixaban, and parenteral agents such as heparin and low-molecular-weight heparins (LMWH), namely enoxaparin [3]. Although they offer therapeutic benefits, anticoagulants present significant challenges due to their narrow therapeutic index, variable pharmacokinetic profiles, high probability for drug–drug interactions (DDIs) with co-administered drugs, and dietary components necessitating the need for therapeutic drug monitoring such as bleeding or thrombosis [4].

Among the anticoagulants, warfarin requires frequent monitoring of the international normalized ratio (INR) to ensure therapeutic efficacy while reducing the risk of bleeding. In contrast, DOACs are characterized by predictable pharmacokinetics which generally do not require routine monitoring despite their use carrying certain risks [5,6]. Researchers have demonstrated that adverse events related to anticoagulants are a major risk factor for hospital admissions and patient morbidity [4,7]. Hence, incorrect prescription of anticoagulants, polypharmacy, and the risk for drug-related problems remains a critical concern in clinical practice, particularly in hospitals providing secondary care where patients diagnosed with comorbidities require multidimensional treatment regimens [8].

Polypharmacy, defined as the prescription containing multiple medications for the management of polymorbidities. is a well-known contributor for the development of undesirable drug effects, DDIs, medication non-compliance, and poor therapeutic outcomes [9,10]. Primarily, advanced-age populations are at higher risk of polypharmacy due to the widespread presence of polymorbidities such as cardiovascular diseases, diabetes mellitus, rheumatological, neurological, and mental health problems requiring the use of numerous medications [11]. So, managing anticoagulant therapy becomes sophisticated in polypharmacy as many co-prescribed medications interact with anticoagulants, tweaking their pharmacodynamics and pharmacokinetics [12]. Taking these into account, it is very important to explore the prescribing practices in the context of polypharmacy and DDIs, in particular in a secondary care facility setting where patient care is provided through multidisciplinary care management.

Drug interactions are a major challenge in healthcare facilities as they compromise the safety and efficacy of drug therapy [13]. Interactions of anticoagulants with co-administered drugs such as antibiotics, antiplatelets, and antifungal drugs can either potentiate the effect, leading to an increased risk of bleeding or diminish their effectiveness, increasing the risk for thromboembolic events [14,15]. Drug interactions must be considered across all stages of drug prescribing, dispensing and administration, as they can also occur throughout the different phases of the pharmacokinetic process. The risk of drug interactions is further compounded by changes in renal and hepatic profile and other clinical variables that may affect drug absorption and elimination [16]. Most drugs, including anticoagulants, are metabolized by the liver enzymes, cytochrome p450 system. Drugs that induce or inhibit these enzymes alter the plasma concentration, thus increasing the potential for adverse outcomes [13,17].

In a hospital setting, the possibility of drug interactions is very high due to the presence of polypharmacy. This is very common with drugs that affect coagulation pathways, such as antiplatelet agents, non-steroidal anti-inflammatory drugs (NSAIDs), and other cardiovascular drugs [8,18]. By focusing on prescribing practices, polypharmacy, and drug interactions in anticoagulant therapy, the study strives to analyze how these factors contribute to patient outcomes in a healthcare setting. The study findings may also help healthcare providers better understand the risk associated with polypharmacy and the need for a systematic approach to managing anticoagulants, particularly in polypharmacy and drug interactions, which will enhance the quality of care in routine clinical care. So, the goal of this research is to look at the prescribing practices, the number of patients on several medications, and the possible interactions between those medications in patients getting anticoagulant therapy in a hospital that provides secondary care.

## 2. Materials and Methods

### 2.1. Study Design and Study Setting

A cross-sectional observational study was undertaken in the outpatient medicine and cardiology department for six months (January 2023–June 2023) in a secondary care hospital in Ras Al Khaimah, U.A.E.

#### Institutional Review Board Statement

The study was conducted in accordance with the Declaration of Helsinki, and ap-proved by the institutional [RAKMHSU-REC-190-2020-F-P dated 26 January 2020], and the Regional Research and Ethics Committee of Ras Al Khaimah, UAE [MOHAP/REC/2022/47-2022-F-P dated 21 February 2023].

### 2.2. Sample Size and Sampling Technique

The study did not rely on a pre-calculated sample size. Instead, all consecutive patients who received at least one anticoagulant prescription and met the inclusion criteria during the six-month study period were included. Therefore, the final sample of 130 patients represents the complete population of eligible anticoagulant users attending the outpatient medicine and cardiology clinics of the hospital during this timeframe. As a census approach was used, no sampling technique was applied.

### 2.3. Criteria for Inclusion and Exclusion

Patients who were at least eighteen years old regardless of their gender were eligible to be included, had an anticoagulant prescription, visited the outpatient clinic of the secondary hospital, and were willing to participate after signing the informed consent form. Individuals who were prescribed anticoagulant therapy without concomitant drugs or had incomplete medical data were not considered for this study.

### 2.4. Study Procedure

Prescription records of all the individuals satisfying the eligibility criteria were considered for the study. In the process of review, patient demographic details, clinical investigations and drugs prescribed to each patient were extracted from the electronic medical records (Wareed system) and recorded in the data recording sheet. All the case records were collected prospectively, and drug therapy was screened for the anticoagulants prescribing practice and co-administered medications.

#### 2.4.1. Assessment of Polypharmacy

We looked at how many drugs were prescribed in each prescription for the study populations at the outpatient pharmacy department. There is no single universally accepted definition of polypharmacy; however, it is commonly defined as the concurrent use of five or more medications. Based on established pharmacoepidemiologic literature, prescriptions were categorized as non-polypharmacy (<5 medications), polypharmacy (5–9 medications), and hyper-polypharmacy (≥10 medications), a threshold previously used to denote excessive medication burden and higher clinical risk [10,19].

#### 2.4.2. Assessment of Drug–Drug Interactions

In order to search for possible DDIs among the prescriptions for anticoagulants, every drug in the patient’s medication profile except those prescribed as ‘stat’ or ‘when necessary/required’ were entered one by one into the ‘drugs to check’ list and were examined using the DrugReax^®^ system from Thomson Reuters Micromedex^®^ [20]. Duplicate interaction pairs arising from identical drug combinations within the same prescription were identified and counted only once. Drugs that were not consistently documented in the electronic medical records, such as over-the-counter and herbal medications, were not included in the analysis. The database contains all interacting combinations captured in the medication profile. Then, the incidence rates and clinical outcomes of these DDIs were measured. The software also provides information about the ‘severity’, ‘onset’, and ‘levels of documentation’ about each pDDIs identified in the study. They were also put into groups based on numerous factors, like ‘severity’ (minor, moderate, major), ‘time of onset’ (rapid, delayed, not specified), and ‘levels of documentation’ (excellent, good, fair, and unknown). The following Micromedex^®^ DrugReax^®^ system definitions of ‘onset’, ‘severity’, and ‘documentation’ were incorporated for deployment in the research.

#### 2.4.3. Criteria for Onset

The severity of an interaction’s repercussions depends on how quickly its clinical effects can materialize and how quickly preventive actions must be taken to avert them. It is categorized as ‘Rapid: The effects will become noticeable 24 h after the interacting medications are administered’ or ‘Delayed: After concurrent administration, the effects will not be noticeable for 24 h’. After concurrent administration, effects might not show up for days or weeks.

#### 2.4.4. Criteria for Severity

The severity level of pDDIs is critical while weighing the risks and benefits of treatment and alternative therapies. The adverse effects of many interactions can be prevented by changing the administration schedule or adjusting the dosage appropriately. There are five levels of severity named as follows: ‘Contraindicated—The drugs are contraindicated for concurrent use’. ‘Major—The interaction may be life-threatening and/or require medical intervention to minimize or prevent serious adverse effects’. ‘Moderate—The interaction may exacerbate the patient’s condition and/or require an alteration in therapy’. ‘Minor—The interaction would have limited clinical impact. Manifestations may include an increase in the frequency or severity of side effects but generally would not require a major alteration in therapy’. ‘Unknown—No known drug interactions’.

#### 2.4.5. Criteria for Documentation

The documentation establishes the level of assurance that an interaction can lead to a changed clinical response. The editorial group’s assessment of the caliber and therapeutic applicability of the main body of research proving the existence of an interaction is reflected in this rating. Nonetheless, many variables may affect whether a patient experiences a well-recorded interaction. The documentation is unaffected by the interaction’s possible intensity of effect and does not mention the interaction’s incidence or frequency. It is categorized as follows: ‘Excellent—Controlled studies have established the existence of the interaction’. ‘Good—Documentation suggests that interactions exist, but well-controlled studies are lacking’. ‘Fair—Available documentation is poor, but pharmacologic considerations lead clinicians to suspect the interaction exists or that documentation is good for a pharmacologically similar drug’. ‘Unknown—Unknown’.

#### 2.4.6. Charlson Comorbidity Index

The Charlson Comorbidity Index (CCI) score is a simple and useful way to figure out how likely it is that someone would die from more than one disease at the same time. It is frequently employed as a mortality and prognostic predictor [21]. The CCI is the most established and reliable technique for estimating death rates. It does this by grouping together illnesses that affect the heart, metabolism, kidneys, liver, lungs, and cancer. Age and the 17 medical comorbidities that may affect the CCI score are combined to create the age-adjusted CCI(CCI), which is between 1 and 6 based on the condition. The CCI’s overall rating is just the sum of the weights, and higher rating reflect more severe comorbidity or multimorbidity.

#### 2.4.7. CHA2DS2-VASc

It is a clinical technique that doctors use to figure out how likely it is that a person with atrial fibrillation (AF) will have a stroke. It assists the prescribers in guiding decisions on whether anticoagulation therapy is required for the prevention of stroke. There is one point for each of the following: being female, being between the ages of 65 and 74, having hypertension, congestive heart failure, diabetes mellitus, or vascular disorders. People 75 years or older who have had a cerebrovascular accident, a transient ischemic attack, or systemic thromboembolism will get two extra points. Clinical decisions on anticoagulant therapy are determined based on the score, which implies a higher risk of stroke. A score of two or higher usually indicates that anticoagulation is necessary.

#### 2.4.8. HAS-BLED Score

It lets doctors figure out how likely it is that patients on anticoagulant therapy may bleed, which leads to safer treatment plans. The scores are based on the following factors: high blood pressure, poor kidney or liver function, stroke, a history of major bleeding or a tendency to bleed, a labile internalized normal ratio (INR), being over 65 years old, taking medications that make you more likely to bleed, and a history of drinking alcohol. A score of 3 or above means a higher risk of bleeding, which is highly essential when evaluating the chances of bleeding from anticoagulation against the chance of stroke.

#### 2.4.9. Data Analysis

The collected data were summated and entered into the Microsoft Excel sheet. IBM, Armonk, New York’s Statistical Package for the Social Sciences (SPSS) version 29.0 for Windows was used to analyse the data. We employed descriptive statistics to summarize the socio-demographic data and showed them as percentages, frequencies, means, and standard deviations (SD). We used binary logistic regression to look at the parameters that are linked to polypharmacy. Bivariate and multiple regression analysis were used to look at the factors that affect the number of DDIs. Age, gender, comorbidities, quantity of medications, and CCI were the predictor variables in this study. They were chosen based on previous research and the availability of data [22,23,24]. To compensate for the confounding variables, the multivariate linear regression analysis included variables with bivariate (*p* < 0.2). The association between continuous factors and the number of pDDIs was estimated using a Pearson correlation. A *p*-value of less than 0.05 was seen to be statistically important.

## 3. Results

### 3.1. Socio-Demographic Characteristics of the Study Populations

In this study of 130 patients, gender-wise distribution showed female prevalence comprising 58.46% of the study population, with the majority in the age range 61–90 years (63.07%). Most participants were not working (62.30%), married (93.84%), and were predominantly UAE nationals (45.38). More than 90% of the study populations had comorbidities, with the highest (37.69%) reporting 3–4 comorbidities. A minor percentage (6.15%) of the study population had no comorbidities. Atrial fibrillation (66.15%) was the most common diagnosis for prescribing anticoagulant therapy in the study populations, followed by deep vein thrombosis (20.76%).

Most participants (71.5%) had a CHA2DS2-VASc score of ≥ 2, indicating a high risk for thromboembolic events. The average CHA2DS2-VASc score was 3.16 ± 1.93, suggesting a generally high risk in the study populations. For bleeding risk, 41.53% were placed under the high-risk category (HAS-BLED score 3–5), with an average score of 2.22 ± 1.33. Regarding comorbidities, 40% of participants had a high CCI (≥5), and 30% had moderate comorbidities (CCI 3–4).

The mean number of medications in the cohort was 8.95 ± 4.73 (range: 2–18) per prescription, with the majority receiving 6–10 medications per prescription. Among the anticoagulants, apixaban (51.53%) was prescribed in more than half of the study populations, and rivaroxaban (33.07%) was prescribed in one-third of the study populations. Warfarin was the least prescribed (6.92%) among the study populations (Table 1).

### 3.2. Types of Comorbidities

**Figure 1:**
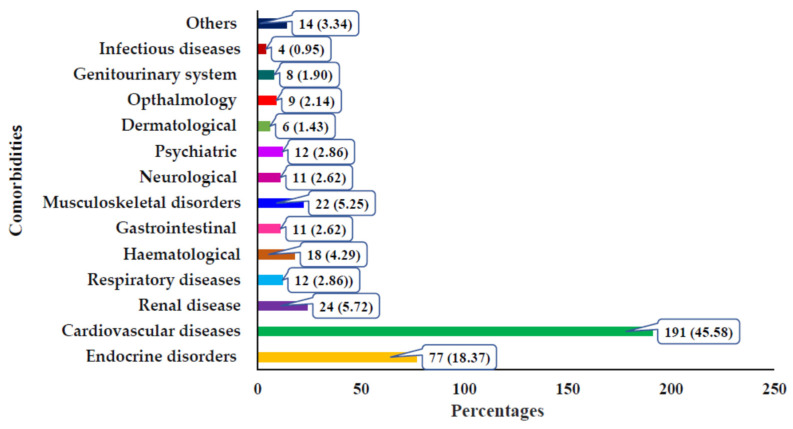
Types of comorbidities among the study populations.

In total, 419 comorbidities were found among the cohort. Among them, the most common being the cardiovascular diseases (45.58%), followed by endocrine disorders (18.37) and renal diseases (5.72%). Neurological diseases and psychiatric disorders accounted for 2.62% and 2.86% of the total comorbidities, respectively (Figure 1).

### 3.3. Class of Medications Received by the Study Populations

**Figure 2:**
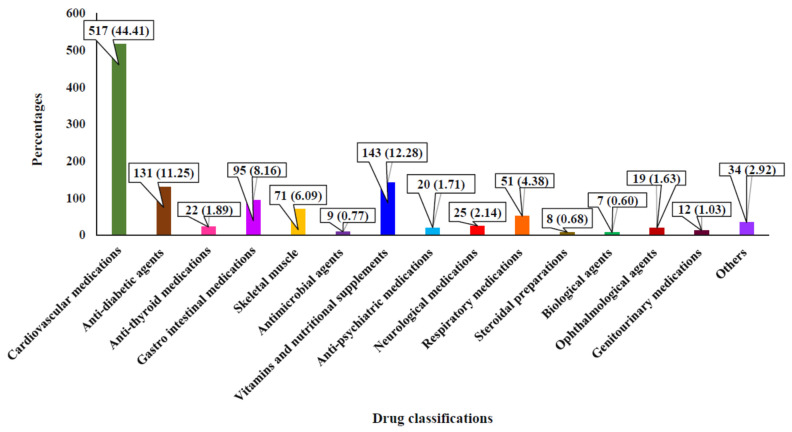
Different class of medications received by the study populations.

In total, 1164 medications from different classes were prescribed among the study populations. The most primarily prescribed were cardiovascular medications (44.41%), followed by vitamins and nutritional supplements (12.28%) and antidiabetic medications (11.25%). Gastrointestinal drugs accounted for 8.16% of the total number of the medication prescribed (Figure 2).

### 3.4. Prevalence of Polypharmacy Among Study Population

Among the total medications received by the study populations, 20% of the patients were prescribed up to 4 or fewer medications, whereas 49 cases (37.69%) were prescribed five to nine medications, which is defined as polypharmacy, and 55 cases (42.30%) were prescribed ten or more drugs, which is categorized as hyper polypharmacy.

### 3.5. Relationship Between Polypharmacy and Demographic, Disease, and Treatment-Related Factors

Polypharmacy was significantly associated with several variables. Age was strongly related with polypharmacy, with those aged 61–90 years more likely to experience polypharmacy (χ^2^ = 31.682, *p* < 0.001). However, there was not a big difference between genders. (χ^2^ = 0.958, *p* = 0.377). Polypharmacy was also more common among UAE nationalities (χ^2^ = 13.947, *p* = 0.003). Comorbidities such as diabetes (χ^2^ = 15.955, *p* < 0.0001), hypertension (χ^2^ = 26.934, *p* < 0.0001), renal disease (χ^2^ = 6.109, *p* = 0.024), and musculoskeletal disorders (χ^2^ = 4.889, *p* = 0.044) were more prevalent with polypharmacy. Additionally, a CCCI score of ≥5 (χ^2^ = 17.700, *p* < 0.0001) was significantly associated with polypharmacy (Table 2).

### 3.6. Factors Associated with Polypharmacy

The logistic regression findings indicated that younger age (OR = 6.76, 95% CI: 2.11–21.66; *p* = 0.001), CCI score (OR = 22.39, 95% CI: 1.15–433.00; *p* = 0.040), and the presence of comorbidities (OR = 30.97, 95% CI: 2.81–341.12; *p* = 0.005) were significantly associated with polypharmacy. However, nationality did not correlate substantially with polypharmacy (OR = 0.426, 95% CI: 0.130–1.395; *p* = 0.159). These findings underscore that age and comorbidities are important factors influencing polypharmacy (Table 3).

### 3.7. Prevalence of pDDIs

**Figure 3:**
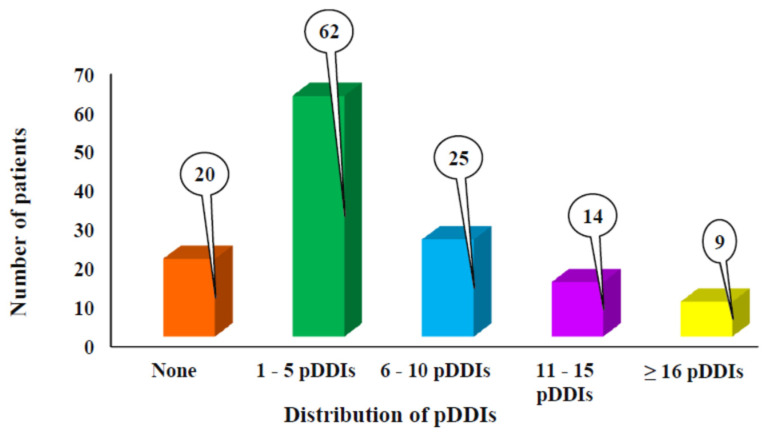
Distribution of pDDIs among the study populations.

Among the study populations, pDDIs were found in 110 out of 130 study patients, resulting in a prevalence of 84.61%. The Micromedex database resource identified 766 pDDIs and 414 interacting drug pairs. The average number of pDDIs was 5.70 ± 6.53 per prescription (range 1–30), with the majority of the patients having 1–5 pDDIs per prescription. Furthermore, 20 prescriptions (15.38%) did not show any potential drug–drug interactions (pDDIs), indicating that pDDIs were absent in a few of the prescribed regimens (Figure 3).

Of the 766 pDDIs identified from 414 Interacting pairs, 120 pDDIs involved anticoagulants from 58 drug combinations. Apixaban [56 (46.66%)] was the most common, followed by rivaroxaban [33 (27.5%)] and warfarin [22 (18.33%)]. Dabigatran was involved in [9 (7.5%)] of the total anticoagulant pDDIs.

### 3.8. Drug Pairs Involved in pDDIs

Among the total 766 pDDIs observed in the study, metformin [157 (20.49%)], bisoprolol [84 (10.96%)], aspirin [59 (7.70%)], and amiodarone [44 (5.74%)] were the most prevalent ones involved drugs causing pDDIs. The most frequently observed drug pairs causing pDDIs were bisoprolol with metformin [15 (1.95%)], apixaban with levothyroxine [14 (1.82%)], Ferrous fumarate—Calcium/cholecalciferol [12 (1.56%)] and atorvastatin with vitamin b complex [11 (1.43%)]. The following table highlights some of the drug pairs involved in drug interactions, highlighting the frequency, severity, documentation quality, onset, and effects of these interactions (Table 4).

### 3.9. Categorization of Drug–Drug Interactions Based on Severity, Onset, and Documentation

The severity, onset, and documentation of pDDIs varied across the study. Among the total 766 pDDIs observed in our research, most of the drug interactions were categorized as ‘moderate’ (52.39%) in level of severity followed by ‘major’ (44.77%). Considering the 120 pDDIs involving anticoagulants, 79 (65.83%) pDDIs were classified as “major” and 41 (34.16%) pDDIs were ‘moderate’ according to the severity level. Based on the onset, most of the pDDIs were documented as “not specified” (68.92%), followed by “delayed” (24.54%) and ‘rapid’ (6.52%), irrespective of their level of severity. However, regarding the documentation of pDDIs, a substantial number are categorized as “fair” (60.31%) and “good” (34.72%) (Table 5).

### 3.10. Relationship Between the Frequency of pDDI and the Quantity of Medications Prescribed

As the number of drugs in the prescription increases, a proportionate increase in the number of pDDIs was also observed, with 100% having interactions in the prescription containing 10 or more medications. The linear-by-linear association between the number of drugs and pDDIs showed statistically significant (χ^2^ = 60.815, *p* < 0.0001), indicating a clear relationship between polypharmacy and increased risk of interactions (Table 6).

### 3.11. Relationship Between Potential Drug–Drug Interactions and Treatment Characteristics

The correlation analysis revealed significant relationships between treatment-related variables and pDDIs. Variables such as age (r = 0.252, *p* = 0.004), total number of drugs (r = 0.388, *p* < 0.0001), CCI (r = 0.358, *p* < 0.0001), and comorbidities (r = 0.534, *p* < 0.0001) showed strong positive correlation with pDDIs (Table 7).

### 3.12. Factors for the Occurrence of pDDIs

The association of pDDIs with treatment-related variables was examined through both bivariate and multivariate analyses. In the bivariate analysis, variables such as male gender (OR = 3.125, 95% CI: 1.153–8.469; *p* = 0.027), age (in years) (OR = 4.238, 95% CI: 1.564–11.488; *p* = 0.005), higher number of medications (<5 medications: OR = 28.963, 95% CI: 7.681–109.211; *p* < 0.001), CCI score (<5) (OR = 16.424, 95% CI: 2.124–127.005; *p* = 0.001) and the presence of comorbidities such as diabetes mellitus (OR = 18.321, CI: 2.370–141.656; *p* < 0.001), and hypertension (OR = 2.852, 95% CI: 1.080–7.529; *p* = 0.040) were significantly associated with pDDIs. According to multivariate analysis, the number of medications remained the only significant predictor (OR = 30.514, 95% CI: 4.056–229.528; *p* = 0.001) for pDDIs (Table 8).

## 4. Discussion

Researchers on drug utilization patterns aim to understand the prescribing behavior of medications within specific populations, thereby providing an important insight into the quality of healthcare and the effectiveness of treatment [25]. This study mainly focused on prescription trends, polypharmacy, and pDDIs in patients receiving anticoagulant pharmacotherapy for thromboembolic and cardiovascular conditions. Gender analysis observed a higher prevalence of female participants than males. This disparities in populations undergoing anticoagulant medication have also been observed in previous studies; however, no credible evidence for gender differences, which may be attributed to physiological and pharmacological reasons [26,27,28,29]. Notably, female is given a point on the CHA2DS2-VASc and HAS-BLED scores because they are more likely to experience thromboembolic events (such as stroke) and bleeding issues, particularly when atrial fibrillation and anticoagulant medication are present.

The majority of participants receiving anticoagulant therapy were aged 60 years and above, a group commonly referred to as the advanced-age population. This observation is in line with the studies from China, Korea, and Belgium, which have reported that most patients receiving anticoagulant therapy are older adults [27,30,31]. This is probably because the risk of thromboembolic events and cardiovascular conditions like heart disease or atrial fibrillation increases with advancing age, necessitating anticoagulant pharmacotherapy to prevent complications like stroke. In our study, UAE nationals were the predominant group receiving anticoagulant drug therapy. A recent study has reported the increased prevalence of cardiometabolic risk factors like obesity, hypertension, diabetes, and dyslipidemia are highly prevalent in a sample of young Emiratis. These factors increase the likelihood of thromboembolic events like strokes or deep vein thrombosis. These elements probably contribute to UAE citizens’ increased demand for anticoagulant medication [32].

Comorbidities were observed in more than 90% of patients with many having three or more. Cardiovascular diseases were the most common condition, consistent with other studies [2,33,34]. A combination of environmental variables, risk factor accumulation, and natural aging is responsible for the greater prevalence of comorbidities in aging populations [35]. A comprehensive multidisciplinary approach along ongoing monitoring is needed to manage this chronic illness that will help to delay the complications and improve quality of life. Atrial fibrillation was the principal reason for anticoagulant medication in our cohort, underscoring the condition’s high frequency and important role in thromboembolic events, particularly strokes. These findings align with preceding investigations on the patterns of anticoagulant usage in healthcare settings [27,29,30].

Being categorized as high risk as per the CHA2DS2-VASc score indicates that majority of participants in our cohort had multiple risk factors for stroke. This reinforces the significance of anticoagulant pharmacotherapy for effectively reducing stroke risk. Compared to the studies from the USA, our results were slightly greater than one but lower than those of another study, indicating that patients using anticoagulant therapy may have different risk profiles [36,37]. The moderate bleeding risk indicated by the HAS-BLED score suggests that anticoagulant medication requires close monitoring. Furthermore, our study sample demonstrated a high comorbidity burden with a mean CCI score of 3.7, which can make the disease management more challenging. In contrast, studies from Belgium and Thailand showed mean CCI values of 4.4 and 6.6, respectively [27,29]. These observations underscore the significance of personalized treatment and monitoring plans tailored to multiple risk factors and comorbidities.

The distribution of anticoagulants in your study indicates a strong preference for apixaban and rivaroxaban, most likely because of their safety profiles, ease of use, and lack of need for regular monitoring, which benefits patients and healthcare professionals alike. It was observed that apixaban was prescribed in our study for indications that involve cardiovascular and thromboembolic events. Identical findings shadowing our results were reported from studies conducted worldwide [33,38,39,40]. Among the DOACs, apixaban has been preferred due to its better medication adherence, medication possession ratio, higher persistence rates, and decreased risk of serious bleeding, as reported in most trials [30,40,41]. Furthermore, apixaban was administered for atrial fibrillation patients with cirrhosis, thrombocytopenia, coagulopathy, CKD/ESRD, and across different CHA2DS2-VASc groups, dementia/cognitive impairment according to few other studies [29,37,42]. These results show that apixaban is gaining popularity among healthcare practitioners over warfarin and other DOACs.

Furthermore, researchers from China and the northern region of the Netherlands have reported a higher frequency of use of rivaroxaban in patients with atrial fibrillation. The possible reason could be its once-daily dosing and broad range of indications compared to other DOACs, which makes it a versatile option for treating different conditions related to thromboembolic risk [31,43]. Dabigatran’s lesser prevalence, however, might be explained by the need for renal monitoring or possible gastrointestinal adverse effects of the drug [44]. The lower utilization of warfarin observed in our study may reflect a shift towards direct oral anticoagulants (DOACs), with warfarin previously serving as the standard anticoagulant. This is probably because warfarin is less convenient than DOACs. After all, it requires regular INR testing, has dietary limitations, and may interfere with other medications.

Numerous studies that have seen an increase in trends in the prescription of DOACs (direct oral anticoagulants) over warfarin with encouraging outcomes further support this evidence [27,33,34,45]. Transient switching has also been reported in a few studies from warfarin to DOACs during the treatment, considering its wider popularity and beneficiaries for atrial fibrillation and prevention and treatment of venous thromboembolism. These studies demonstrate the growing popularity of DOACs over warfarin because of their simplicity, consistent pharmacokinetics, and lack of need for frequent monitoring, lower rates of hemorrhagic stroke and mortality, all of which enhance patient adherence and therapeutic outcomes [2,36]. Additionally, clinical guidelines also recommend apixaban as a first-line treatment for thromboembolic events, particularly for stroke prevention, which may explain its higher use in our cohort [46].

Polypharmacy was observed in a higher proportion of cohorts with medications related to the cardiovascular system being the most prevalent. Contrary to our findings, studies related to cardiovascular diseases from Ghana and India reported a mean number of 8.92 and 11.7 drugs per prescription respectively [2,45]. The increase prevalence of polypharmacy in our cohort is likely driven by the greater burden of co-existing comorbidities. The study observed that age, nationality, CCI, and comorbidities such as diabetes mellitus, hypertension, renal diseases, and rheumatological diseases are significant risk factors associated with polypharmacy. Similar observations were reported in previous studies, which highlighted that polypharmacy is more common among patients with chronic diseases [47,48]. It is evident that managing complex health conditions, particularly in aging populations, often necessitates polypharmacy; however, the potential risks associated with multiple medications must be carefully balanced against the benefits to ensure optimal treatment outcomes. Furthermore, an increase in the number of comorbidities often predisposes patients to polypharmacy, which in turn is a known risk factor for medication non-adherence and drug-related problems that can further complicate disease management [49]. Therefore, a comprehensive review of drug therapy with regular monitoring, and avoiding unnecessary prescriptions are crucial in mitigating the risk of polypharmacy, particularly in aging populations with multiple comorbidities and those on anticoagulant therapy.

Drug–drug interactions (DDIs) represent a significant challenge in modern medicine, particularly as polypharmacy becomes more prevalent, especially among older adults. Since patients usually receive different drug combinations in real-world clinical settings, DDI identification and assessment are essential for safeguarding patient well-being and maximizing therapeutic efficacy [50]. The prevalence of pDDIs observed in the present study is comparable to the wide range reported in previous studies (21.1–99.2%) [51,52,53].

The variation in prevalence can be attributed to differences in study population characteristics, data collection methods, healthcare settings, periods, and definitions used to assess pDDIs. Acknowledging these factors can provide a clearer understanding of why prevalence rates might differ across studies. The high prevalence in our study could be associated with polypharmacy. Among the total pDDIs observed in our study, a notable proportion of interactions included anticoagulant medications. Our findings indicate that pDDIs involving anticoagulants commonly occurred when these agents were co-prescribed with antiarrhythmics, antiplatelets, statins, NSAIDs, diuretics, proton pump inhibitors, antiepileptics, and psychiatric medications.

Research has shown that the prescription of these drugs, either separately or in combination, is the cause of a number of interactions [54]. According to our data, 95% of patients who are taking anticoagulant medications that interact are at higher risk of bleeding. The observed outcomes are likely mediated through several pharmacodynamic and pharmacokinetic mechanisms. When amiodarone and rivaroxaban are administered together, they interact through the inhibition of liver enzymes, particularly the CYP450 system, which can increase the plasma levels of rivaroxaban, leading to an enhanced anticoagulant effect and higher bleeding risk. Similarly, apixaban and aspirin both increase the risk of bleeding, as aspirin’s antiplatelet effect combines with apixaban’s anticoagulant properties, amplifying bleeding tendencies.

The pDDIs between apixaban and celecoxib or escitalopram may be due to enzyme inhibition or altered platelet function, further raising the risk of hemorrhage. Administration of statins, such as rosuvastatin and atorvastatin, can increase anticoagulant exposure by affecting the metabolism of anticoagulants like warfarin and dabigatran, likely via CYP450 interactions, which may lead to an elevated INR and increased bleeding risk. Additionally, coadministration of levothyroxine and apixaban or rivaroxaban may interact through changes in the metabolism or absorption rates of anticoagulants, altering their effectiveness and risk of bleeding. These interactions emphasize the importance of regular monitoring of drug levels, renal function, and bleeding risk when prescribing multiple medications concurrently, especially in patients on anticoagulant therapy.

Of the many possible pDDIs detected using the Micromedex database, some alerts have no real clinical significance. The databases that report interactions are designed to be as sensitive as possible. They might cite interactions involving hypothetical reasoning, drug mechanisms and crafting, or in some cases, a single report which have no bearing on clinical practice. As a result, some drug pairs that are common in this study, such as atorvastatin calcium and vitamin B complex, have no clinical significance in the prescribing and drug interaction information and literature. Additionally, numerous common drug pairs may be the appropriate and prescription-compliant co-morbidities in cardiovascular disease to limit the cardiovascular disease and associated comorbidities. The benefits of therapy may exceed the risks, especially when monitoring is appropriate. Potentially harmful drug interactions may indicate a true lack of evidence of risk when evaluated more broadly in the clinical ranking context.

Drug–drug interactions (pDDIs) involving non-anticoagulant medications that can significantly impact clinical outcomes are also observed in this study. These medications’ interactions may lead to various adverse effects, affecting multiple organ systems. These include changes in blood glucose levels, electrolyte imbalances, cardiovascular effects, liver toxicity, gastrointestinal and renal issues, drug efficacy and toxicity, and increased risk of bleeding or seizures. These medications are prescribed more frequently in our cohort, and prompt attention to these potential interactions could help prevent adverse consequences. These drugs are medically useful and can save lives, but prescribing them is occasionally inevitable. Consequently, careful observation and modification of treatment plans when pDDIs are suspected is essential to mitigate adverse outcomes. Furthermore, a thorough benefit-risk assessment of prescribed medications is warranted to ensure safety and efficacy.

In our research, pDDIs were largely moderate to major in severity, underscoring their potential clinical significance. Previous studies reported varying levels of severity, with some studies finding most pDDIs to be major, while others reported a higher prevalence of moderate and minor pDDIs [52,55,56,57]. The discrepancy in severity across studies can be due to differences in the populations studied, clinical practices, methods used to identify and categorize interactions, and the healthcare settings. Furthermore, among the pDDIs involving anticoagulants, most were classified as major, followed by moderate. To reduce risks, including bleeding, in patients using anticoagulants, our findings highlight the significance of closely monitoring anticoagulant medication, quickly discovering interactions and modifying treatment strategies.

The study observed that in line with earlier studies, drug–drug interactions (DDIs) were substantially correlated with age, comorbidities, number of prescriptions, and CCI(CCI) but not with gender [53,55,58].

Age-related physiological changes and the requirement for several drugs to control comorbidities raise the risk of DDIs in aging populations, increasing the chance of drug-related issues and polypharmacy [58,59]. Strong correlations were found between the risk of DDIs and the CCI, which measures the severity of comorbid diseases. A higher number of medications in the prescription significantly increased the risk of potential drug–drug interactions, aligning with findings from a Brazilian study [60].

The number of drugs prescribed emerged as a significant predictor of pDDIs in both bivariate and multivariate analyses. pDDIs were more common among patients taking five or more drugs, especially those with complicated drug regimens. Anticoagulants, vital in treating cardiovascular and thromboembolic events, are often prescribed alongside other medications for patients with comorbidities, such as cardiovascular disease, diabetes, and renal issues [61]. This raises the possibility of moderate-to-severe medication interactions, which could result in problems like bleeding or decreased effectiveness of anticoagulants, as reported in our study. In older populations, where polypharmacy is commonly used, this problem is particularly prevalent. Continuous monitoring, dose modifications, cautious medication selection, avoiding needless combinations, and the use of clinical decision support systems (CDSS) are crucial for maximizing therapeutic outcomes with anticoagulant medications, thereby reducing the risks.

This study identified the most clinically relevant potential drug–drug interactions, or pDDIs, concerning anticoagulants, and the concomitant use of antiplatelet agents, antiarrhythmics, statins, NSAIDs, and antidiabetics. These interactions are primarily due to the pDDIs being caused by the drugs involved and the pDDIs being caused by the drugs involved due to the actions of the cytochrome P450 system and p-glycoprotein, and the pharmacodynamic effects due to excessive or intensified anticoagulant effects, which lead to increased risks of haemorrhage. The predominance of moderate, major, and moderate to major pDDIs speaks volumes to the clinically relevant pDDIs especially pertaining to the elderly, or the patients, with polypharmacy and multiple concomitant comorbid conditions. Comprehensive management of these pDDIs will undoubtedly include, but not be limited to, regular comprehensive medication reviews, complete avoidance of the identified or suspected “high-risk” medication combinations, careful dose titrations, and the use of “high” monitoring parameters. Additionally, real-world studies have been undertaken to validate this, and although the evidence indicates that DOACs are comparatively safer, it has also been shown that drug interactions will, and most often, increase the risk of undesirable outcomes [62].

### 4.1. National Insights from UAE Anticoagulant Studies

Several studies from the United Arab Emirates (UAE) have investigated various aspects of anticoagulant use, particularly in patients with non-valvular atrial fibrillation (NVAF) [63,64,65]. Earlier research focused on warfarin revealed challenges such as suboptimal INR control, limited patient awareness, and the need for individualized dosing strategies, including pharmacogenomics-based approaches [66]. Surveys conducted among pharmacists highlighted moderate levels of knowledge and suboptimal counselling practices, especially in community settings [67]. Registry and real-world claims database analyses—such as the FLOW-AF Registry and the Dubai Real-World studies—have primarily focused on trends in anticoagulant prescribing, cost implications, treatment adherence, clinical monitoring, and one-year outcomes. These studies consistently demonstrated a growing preference for DOACs over warfarin, reporting better safety, fewer complications, and greater patient adherence. Some investigations also evaluated healthcare resource utilization and the economic burden of anticoagulant therapy in the UAE [63,64,65,68,69]. However, these studies were largely retrospective or registry-based and lacked detailed assessments of clinical safety risks arising from complex medication regimens.

In contrast, the present study provides novel, practice-relevant evidence by employing a prospective design based on real patient-level data from a public hospital in the UAE. This study is the first to specifically evaluate the burden of polypharmacy and pDDIs in patients receiving anticoagulant therapy, using standardized interaction classification tools and linking clinical findings with validated risk scores such as HAS-BLED, CHA_2_DS_2_-VASc, and Charlson Comorbidity Index (CCI). Given the UAE’s high prevalence of obesity, cardiovascular disease, and diabetes, which often necessitate complex multidrug regimens, the study highlights the significant safety risks posed by polypharmacy and unrecognized pDDIs in anticoagulated patients.

Given the UAE’s high burden of cardiovascular disease, diabetes, and obesity—which often necessitate complex pharmacotherapy—the study underscores the significant safety risks associated with polypharmacy and unrecognized pDDIs [32,70]. Unlike previous research, it also proposes system-level interventions, such as the integration of Clinical Decision Support Systems (CDSS) into electronic prescribing platforms to flag high-risk combinations, pharmacist-led medication therapy management (MTM) for deprescribing support, and development of institutional protocols for risk stratification. These recommendations offer practical solutions to enhance the safety and efficacy of anticoagulant therapy and contribute valuable guidance for national policy development.

In summary, this study provides a unique, clinically actionable contribution to UAE literature by bridging the gap between epidemiological patterns and real-world medication safety—supporting optimized, patient-centered anticoagulation management in polymorbid populations.

The strength out study includes having a structured methodology, clearly defined inclusion and exclusion criteria, and the use of validated clinical risk assessment tools. The clinical applicability of the findings is also boosted by the detailed assessment of polypharmacy and pDDIs.

### 4.2. Limitations

Only one database was used to analyse the pDDIs in our investigation, which limited the number and might not have included all of the pDDIs. It might be possible to characterize the results more using comparison and multiple database tools. The current study primarily focused on theoretical pDDIs and employed data gathered from electronic health records without follow up, it was unable to address the real clinical outcomes. Therefore, the actual clinical relevance of certain pDDIs may vary depending on patient-specific and treatment-related factors. Given that the study was observational and depended on prescriptions from electronic health records, it was impossible to gather information about over-the-counter medications, herbal remedies, or dietary supplements, nor could it examine their pDDIs when taking other medications simultaneously. The wide confidence intervals observed for some estimates may reflect the sample size and distribution of events across subgroups. It was a single-centre design although representing all patients who met the study criteria, smaller sample size limits the generatability of the findings. Lastly, the quality of data and depth of understanding of clinical outcomes are impacted by the short observation duration and the lack of demographic information.

## 5. Conclusions

The present study observed a higher prevalence of polypharmacy and pDDIs among the study populations receiving anticoagulant drug therapy. Apixaban was the most commonly prescribed anticoagulant for preventing and treating thromboembolic events. A significant correlation was observed for age, CCI, comorbidities, number of medications, and the risk for pDDIs. The results underscore the importance of a multidimensional framework consisting of careful medication therapy review with deprescribing consideration, tailoring the drug regimen to the individual, patient education, continuous monitoring, regular follow-up, and a multidisciplinary approach between healthcare providers is paramount for effective management. Utilizing a clinical decision support system, evidence-based practice, and the involvement of clinical pharmacists may help address many challenges associated with its use as the number of patients receiving newer anticoagulants over time increased substantially, as noticed in our study. This will help to develop best practices for integrating the management of polypharmacy and multimorbidity, minimizing risks of pDDIs, and improving the quality of care that will enhance health outcomes for these patients.

## Figures and Tables

**Table 1:** Sociodemographic characteristics of the study populations (*n* = 130).

Clinical Characteristics	Frequency (*n* = 130)	Percentage
Gender		
Female	76	58.46
Male	54	41.53
Age (in Years)		
≤30	04	3.07
31–60	42	32.30
61–90	82	63.07
>90	02	1.53
Mean Age (in years)	64.9 ± 17.2	
Nationality		
Asians	26	20
United Arab Emirates (UAE)	59	45.38
Middle Eastern countries other than the UAE	37	28.46
Others	08	6.15
Employment		
Working	45	34.61
Not working	81	62.30
Retired	04	3.07
Marital status		
Married	122	93.84
Unmarried	02	1.53
Widow	05	3.84
Divorced	01	0.76
Comorbidities		
Nil	08	6.15
1–2	41	31.53
3–4	49	37.69
≥5	32	24.61
Current diagnosis		
Atrial fibrillation	86	66.15
Atrial flutter	05	3.84
Deep vein thrombosis	27	20.76
Pulmonary embolism	11	8.46
Mitral valve	01	0.76
Categorization Based on CHA2DS2-VASc Score (*n* = 86)		
Low risk (Score 0)	03	3.48
Moderate risk (Score 1)	10	11.62
High risk (Score ≥ 2)	73	84.88
Average score (range)	3.64 ± 1.90 (0–7)	
HAS-BLED score (*n* = 86)		
0–1: Relatively Low risk	17	19.76
2: Moderate risk	29	33.72
3–5: High risk	40	46.51
Average score (range)	2.37 ± 1.28 (0–5)	
Classification Based on the CCI(CCI):		
CCI = 0: Low Comorbidity	16	12.30
CCI = 1–2: Mild Comorbidity	23	17.69
CCI = 3–4: Moderate Comorbidity	39	30.0
CCI = 5 or more: High Comorbidity	52	40.0
Average score	3.70 ± 2.39	
Number of drugs per prescription		
1–5	26	20.0
6–10	49	37.69
11–15	40	30.76
≥16	15	11.53
Total number of medications	1164	
Average number of medications per patient	8.95 ± 4.73	
Classification of Anticoagulant medications		
Apixaban	67	51.53
Dabigatran	11	8.46
Rivaroxaban	43	33.07
Warfarin	09	6.92

**Table 2:** Bivariate analysis of factors associated with polypharmacy among the study populations.

Variables	Categories	Polypharmacy		χ^2^	*p* Value
		Presence (*n* = 104)	Absence (*n* = 26)		
Gender	Male	41 (39.42)	13 (50.0)	0.958	0.377
	Female	63 (60.57)	13 (50.0)		
Age (in years)	≤30	1 (0.96)	3 (11.53)	31.682	<0.0001
	31–60	24 (23.07)	18 (69.23)		
	61–90	77 (74.03)	5 (19.23)		
	>90	02 (1.92)	00 (0.0)		
Nationality	Asians	16 (15.38)	10 (38.46)	13.947	0.003
	UAE	55 (52.88)	4 (15.38)		
	Middle Eastern countries other than the UAE	28 (26.92)	9 (34.61)		
	Others	05 (4.80)	3 (11.53)		
CCI	<5	80 (76.92)	26 (100.0)	17.700	<0.0001
	≥5	24 (23.07)	0 (0.0)		
Diabetes mellitus	Yes	53 (50.96)	02 (7.69)	15.955	<0.0001
	No	51 (49.03)	24 (92.30)		
Hypertension	Yes	80 (76.92)	06 (23.07)	26.934	<0.0001
	No	24 (23.07)	20 (76.92)		
Ischemic heart disease	Yes	21 (20.19)	01(3.84)	1.795	0.234
	No	84 (80.76)	25 (96.15)		
Renal disease	Yes	24 (23.07)	00 (0.0)	6.109	0.024
	No	80 (76.92)	26 (100.0)		
CVA	Yes	11 (10.57)	00 (0.0)	3.004	0.120
	No	93 (89.42)	26 (100.0)		
Rheumatological diseases	Yes	17 (16.34)	00 (0.0)	4.889	0.044
	No	87 (83.65)	26 (100.0)		
Asthma	Yes	09 (8.65)	01(3.84)	0.677	0.686
	No	95 (91.34)	25 (96.15)		

*p* < 0.05 statistically significant; *p* < 0.01 statistically highly significant, CCI(CCI); Cerebrovascular attack (CVA).

**Table 3:** Multiple binary logistic regression analysis for factors associated with polypharmacy among the study populations.

	Unstandardized Coefficients		Standardized Coefficients	*p* Value	Odds Ratio (95% CI)
	B	Standard Error	Wald		
Age (in years)	1.912	0.594	10.368	0.001	6.765 (2.113–21.658)
Nationality	−0.852	0.605	1.986	0.159	0.426 (0.130–1.395)
CCI	3.109	1.511	4.233	0.040	22.399 (1.159–433.003)
Comorbidities	3.433	1.224	7.868	0.005	30.977 (2.813–341.124)
Constant	−10.711	3.448	9.647		

*p* < 0.05 statistically significant; *p* < 0.01 statistically highly significant, CCI(CCI).

**Table 4:** Most common drug pairs involved in drug–drug interactions.

Drug Pairs	Frequency (%)	Severity	Documentation	Onset	Effect of Drug Interactions
Atorvastatin calcium–Vitamin B Complex	11 (1.43)	Major	Good	Delay	Increased risk of myopathy and rhabdomyolysis.
Amiodarone–Apixaban	8 (1.04)	Major	Good	NS	Increased apixaban exposure and increased risk of bleeding
Apixaban–Aspirin	8 (1.04)	Major	Good	NS	Increased risk of bleeding and can cause serious, potentially fatal bleeding.
Furosemide–Empagliflozin	7 (0.91)	Major	Fair	NS	Increased risk of hyperglycemia and an increased insulin requirement.
Celecoxib–Rivaroxaban	5 (0.65)	Major	Fair	NS	Increased risk of bleeding
Sacubitril/valsartan–Furosemide	08 (1.04)	Major	Fair	NS	Severe hypotension and deterioration in renal function, including renal failure.
Amlodipine–Metformin	7 (0.91)	Moderate	Fair	NS	Increased risk of hyperglycemia and potential loss of glycaemic control.
Bisoprolol–Dapagliflozin	09 (1.17)	Moderate	Good	Delay	Hypoglycemia or hyperglycemia; decreased symptoms of hypoglycemia.
Amiodarone–Atorvastatin	08 (1.04)	Moderate	Good	NS	Increased risk of Myopathy and rhabdomyolysis
Aspirin–Bisoprolol	06 (0.78)	Moderate	Good	Delay	Reduced antihypertensive effect
Furosemide–Metformin	05 (0.65)	Moderate	Fair	NS	Increased risk of hyperglycemia and potential loss of glycemic control.
Bisoprolol–Tamsulosin	05 (0.65)	Moderate	Good	Rapid	Exaggerated hypotensive response to the first dose of the alpha-blocker.
Esomeprazole sodium–Ferrous sulfate	6 (0.78)	Moderate	Fair	NS	Reduced iron bioavailability.
Bisoprolol fumarate-Metformin hydrochloride	15 (1.95)	Moderate	Good	Delay	Hypoglycaemia or hyperglycemia; decreased symptoms of hypoglycemia.
Apixaban–Levothyroxine sodium	14 (1.82)	Moderate	Fair	NS	Increased response to oral anticoagulant therapy.
Clopidogrel hydrogen sulphate–Atorvastatin	4 (0.52)	Moderate	Excellent	NS	Decreased formation of clopidogrel active metabolite resulting in high on-treatment platelet reactivity.
Amiodarone–Rivaroxaban	4 (0.52)	Moderate	Good	NS	Increased rivaroxaban exposure and increased risk of bleeding
Celecoxib–Valsartan	01(0.13)	Moderate	Excellent	NS	Reduced antihypertensive effect renal dysfunction and/or increased blood pressure.
Levothyroxine–Rivaroxaban	3 (0.39)	Moderate	Fair	NS	Increased response to oral anticoagulant therapy.
Atenolol–Calcium carbonate	2 (0.26)	Minor	Good	Rapid	Reduced effectiveness of atenolol.
Ferrous fumarate–Calcium/cholecalciferol	12 (1.56)	Minor	Fair	Delay	Decreased iron effectiveness.
Chondroitin sulfate sodium/glucosamine–Metformin hydrochloride	03 (0.39)	Minor	Fair	Rapid	Reduced antidiabetic agent effectiveness.

Potential drug–drug interactions (pDDIs) were identified using the Micromedex database and do not necessarily indicate clinically significant interactions; clinical relevance should be interpreted in the context of patient-specific factors and therapeutic indications.

**Table 5:** Severity, Documentation, and onset of the detected pDDIs.

Severity	Onset	Documentation			
		Excellent	Fair	Good	Unknown
Minor (*n* = 22; 2.87%)	Delayed	00 (0.0)	13 (59.09)	00 (0.0)	00 (0.0)
	Not specified	00 (0.0)	00 (0.0)	00 (0.0)	00 (0.0)
	Rapid	00 (0.0)	04 (18.18)	05 (22.72)	00 (0.0)
Moderate (*n* = 401; 52.34%)	Delayed	03 (0.74)	17 (4.23)	117 (29.17)	00 (0.0)
	Not specified	13 (3.24)	178 (44.38)	41 (10.22)	00 (0.0)
	Rapid	04 (0.99)	18 (4.48)	10 (2.49)	00 (0.0)
Major (*n* = 343; 44.77%)	Delayed	00 (0.0)	02 (0.58)	36 (10.49)	00 (0.0)
	Not specified	16 (4.66)	227 (66.18)	53 (15.45)	00 (0.0)
	Rapid	02 (0.58)	03 (0.87)	04 (1.16)	00 (0.0)

**Table 6:** Number of drugs prescribed, the frequency of pDDIs, and their linear-by-linear associations.

		Total Number of Drugs in Prescriptions						Total	Linear-by-Linear Associations	
		2–4	5–7	8–10	11–13	14–16	17–20		χ^2^ Value	*p*-Value
Interactions	No	13	6	1	00	00	00	20	60.815	<0.0001 * £
	Yes	7	17	31	30	13	12	110		
	Total	20	23	32	30	13	12	130		

* *p* < 0.05 statistically significant; £-Fischer’s exact.

**Table 7:** Correlation between pDDIs and treatment-related variables.

r (*p*-Value)	Gender	Age	CCI	Total Number of Drugs	Comorbidities	Number of Drug Interactions
Gender		0.147 (0.094)	0.011 (0.903)	0.086 (0.331)	0.108 (0.219)	0.053 (0.547)
Age			0.676 (0.000)	0.474 (0.000)	0.500 (0.000)	0.252 (0.004)
CCI				0.205 (0.000)	0.693 (0.000)	0.358 (0.000)
Total number of drugs					0.582 (0.000)	0.388 (0.000)
Comorbidities						0.534 (0.000)
Number of drug interactions						

Correlation is significant at the 0.01 level (2-tailed), CCI(CCI).

**Table 8:** Association of pDDIs with treatment-related variables.

Variable		Bivariate Analysis		Multivariate Analysis	
		Crude OR (95% CI)	*p*-Value	Adjusted OR (95% CI)	*p*-Value
Gender					
	Male	3.125 (1.153–8.469)	0.027	3.766 (0.958–14.811)	0.058
	Female	Ref.			
Age (in years)					
	<50	4.238 (1.564–11.488)	0.005	1.433 (0.269–7.650)	0.673
	≥50	Ref.		Ref	
Number of medications					
	<5	28.963 (7.681–109.211)	<0.001	30.514 (4.056–229.528)	0.001
	≥5	Ref.			
CCI					
	<5	16.424 (2.124–127.005)	0.001	2.161 (0.188–24.860)	0.536
	≥5	Ref			
Diabetes mellitus					
	Yes	18.321 (2.370–141.656)	<0.001	6.316 (0.579–68.911)	0.131
	No	Ref.		Ref	
Hypertension					
	Yes	2.852 (1.080–7.529)	0.040	0.122 (0.022–1.352)	0.094
	No	Ref.		Ref	
Thyroid disorders					
	Yes	4.483 (0.568–35.398)	0.194	6.059 (0.498–73.695)	0.158
	No	Ref.		Ref	
Rheumatological diseases					
	Yes	3.234 (0.404–25.876)	0.311	--	
	No	Ref			
Renal					
	Yes	3.857 (0.846–17.578)	0.098	2.447 (0.302–19.818)	0.402
	No	Ref		Ref	
CVA					
	Yes	0.731 (0.218–2.456)	0.741	--	
	No	Ref			
IHD					
	Yes	2.757 (0.759–10.018)	0.183	2.499 (0.355–17.600)	0.358
	No	Ref			

## Data Availability

The data supporting the findings of this study are available from the corresponding author upon reasonable request.

## Abbreviations

The following abbreviations are used in this manuscript:

pDDIs	Potential drug–drug interactions
DVT	Deep vein Thrombosis
DOAG	Direct oral anticoagulants
LMWH	Low-molecular-weight heparin
UAE	United Arab Emirates
CCI	Charlson Comorbidity Index
INR	Internationalized Normal Ratio
